# Deleting the IF_1_-like *ζ* subunit from *Paracoccus denitrificans* ATP synthase is not sufficient to activate ATP hydrolysis

**DOI:** 10.1098/rsob.170206

**Published:** 2018-01-24

**Authors:** Febin Varghese, James N. Blaza, Andrew J. Y. Jones, Owen D. Jarman, Judy Hirst

**Affiliations:** The Medical Research Council Mitochondrial Biology Unit, University of Cambridge, Wellcome Trust/MRC Building, Biomedical Campus, Hills Road, Cambridge CB2 0XY, UK

**Keywords:** ADP inhibition, ATP hydrolysis, bioenergetics, *ɛ* subunit, oxidative phosphorylation, reversible catalysis

## Abstract

In oxidative phosphorylation, ATP synthases interconvert two forms of free energy: they are driven by the proton-motive force across an energy-transducing membrane to synthesize ATP and displace the ADP/ATP ratio from equilibrium. For thermodynamically efficient energy conversion they must be reversible catalysts. However, in many species ATP synthases are unidirectional catalysts (their rates of ATP hydrolysis are negligible), and in others mechanisms have evolved to regulate or minimize hydrolysis. Unidirectional catalysis by *Paracoccus denitrificans* ATP synthase has been attributed to its unique *ζ* subunit, which is structurally analogous to the mammalian inhibitor protein IF_1_. Here, we used homologous recombination to delete the *ζ* subunit from the *P. denitrificans* genome, and compared ATP synthesis and hydrolysis by the wild-type and knockout enzymes in inverted membrane vesicles and the F_1_-ATPase subcomplex. ATP synthesis was not affected by loss of the *ζ* subunit, and the rate of ATP hydrolysis increased by less than twofold, remaining negligible in comparison with the rates of the *Escherichia coli* and mammalian enzymes. Therefore, deleting the *P. denitrificans ζ* subunit is not sufficient to activate ATP hydrolysis. We close by considering our conclusions in the light of reversible catalysis and regulation in ATP synthase enzymes.

## 1. Background

F_1_F_O_ ATP synthases are energy-transducing enzymes that use the energy stored in electrochemical proton (or sodium) motive forces across the membranes of bacteria, chloroplasts or mitochondria to generate ATP from ADP and inorganic phosphate [1]. They catalyse by a mechanical rotary mechanism [1–3]. ADP is converted to ATP in the membrane-extrinsic F_1_ domain, driven by conformational changes induced by rotation of the central stalk, which is in turn driven by proton transfer through the membrane-bound F_O_ motor domain. The energy released by dissipating the proton-motive force is thus captured by displacing the ATP/ADP ratio from its equilibrium position. A peripheral stalk acts to prevent the F_1_ domain rotating, without catalysis, together with the central stalk.

Under conditions of low proton-motive force and high ATP/ADP ratio the thermodynamics of the system favour ATP hydrolysis over ATP synthesis, and rotation may reverse to dissipate the energy stored in the high ATP/ADP ratio and build the proton-motive force. Under anaerobic conditions many bacterial ATP synthases hydrolyse the ATP produced by glycolysis to generate a proton-motive force for the support of essential cellular functions [4,5]. However, should the proton-motive force not be usefully employed and lost to proton leak, the hydrolysis reaction is wasteful, and it has been assumed that this explains why many organisms have developed strategies to regulate and prevent it occurring.

**Figure 1.**
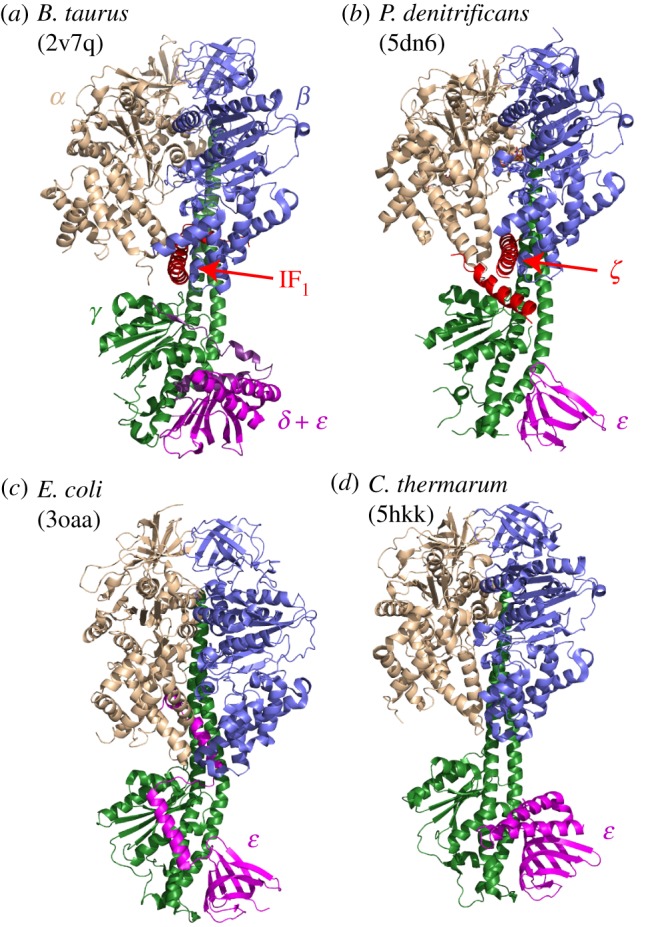
Structural data on four ATP synthase enzymes that do not catalyse ATP hydrolysis. (*a*) *B. taurus* F_1_-ATPase with the inhibitory domain of the inhibitor protein bound [11]. (*b*) *P. denitrificans* ATP synthase containing the *ζ* subunit [13]. (*c*) *E. coli* F_1_-ATPase with the *ɛ* subunit in the ‘up’ state in which ATP hydrolysis is inhibited [14]. (*d*) *C. thermarum* F_1_-ATPase [15].

Three distinct ‘ratchet-like’ mechanisms have evolved to regulate and prevent ATP hydrolysis by ATP synthases. In chloroplasts, an ADP-inhibited state, formed when MgADP remains bound to one of the catalytic sites, is stabilized under dark conditions by formation of an intramolecular disulfide bond, then released under light conditions by thioredoxin-regulated reduction [1,6]. In mitochondria, an inhibitor protein, IF_1_, binds to the ATP synthase during hydrolysis and blocks the rotary mechanism [7,8]. When the proton-motive force increases, rotation in the synthesis direction expels the inhibitor protein, and ATP synthesis resumes [9]. Structures of both the *Bos taurus* (bovine) and *Saccharomyces cerevisae* (yeast) enzymes have been determined with their respective inhibitor proteins bound [10–12], showing that they bind at a catalytic interface between specific α and β subunits and the γ central stalk subunit in F_1_ (figure 1), but also that the catalytic cycles are arrested at different stages in the two cases [1]. In bacteria such as *Escherichia coli* and *Bacillus* PS3, which use ATP synthase to synthesize ATP under aerobic conditions but hydrolyse it under anaerobic conditions, hydrolysis has been proposed to be regulated by the *ɛ* subunit [16] that connects the central stalk to the membrane-bound *c*-ring rotor. The helical C-terminal domain of the subunit adopts two different conformations: the ‘down’ conformation is stabilized by a bound ATP molecule, whereas the ‘up’ conformation has been proposed to inhibit hydrolysis by inserting into a subunit interface in the F_1_ domain [14,17–21] (figure 1). Importantly, all three ratchet mechanisms rely on removing the enzyme from the catalytic cycle by converting it to a stable, off-pathway state. By contrast, some bacterial ATP synthases, such as from *Caldalkalibacillus thermarum* and *Mycobacterium tuberculosis*, are unable to hydrolyse ATP under any physiologically relevant conditions [15,22], but no stable off-pathway states have been identified. Their irreversibility has been linked to a possible altered conformation of the γ subunit [23], but evidence to support this link is lacking. It has also been linked to the C-terminus of the *ɛ* subunit [24], but structural and mutagenesis data on the *C. thermarum* enzyme (figure 1) have questioned this relationship [15]. Instead, it has been proposed that, in *C. thermarum*, hydrolysis is inhibited by product inhibition and extremely slow ADP release. This proposal clearly resembles the inhibition of hydrolysis by ADP that has been widely studied in mammalian and bacterial systems [25–27], although the inhibition is much more severe in *C. thermarum* and if it shares a common origin the effect must be very exaggerated. Thus, the unidirectional ATP synthases from *C. thermarum* and *M. tuberculosis* may be unable to catalyse ATP hydrolysis effectively because they become trapped in an excessively stable on-pathway state, from which they are unable to escape in the hydrolysis direction.

ATP synthase in the bacterium *Paracoccus denitrificans* is a further example of an enzyme that is able to catalyse ATP synthesis rapidly, but unable to hydrolyse it effectively [28]. In this case, the enzyme contains an unusual subunit, the *ζ* (zeta) subunit, which resembles the mitochondrial inhibitor protein [29]. When immunoaffinity chromatography was used to strip the *ɛ* and *ζ* subunits from *P. denitrificans* F_1_-ATPase, subsequent addition of a recombinant form of the *ζ* subunit resulted in a substantial decrease in the sulfite-stimulated rate of hydrolysis [30]. Similar results were not obtained using an N-terminally truncated form, suggesting that the N-terminus forms the inhibitory domain. Subsequently, the structure of the *P. denitrificans* F_1_F_O_ complex, determined by X-ray crystallography at 4.0 Å resolution [13], revealed structural similarities between the interactions of the N-terminus of the *ζ* subunit with the *P. denitrificans* F_1_F_O_ ATP synthase and the interactions of the inhibitory portions of the bovine and yeast IF_1_ proteins with their respective enzymes (figure 1) [10–13]. The *ζ* subunit was therefore proposed to prevent ATP hydrolysis by *P. denitrificans* ATP synthase.

Here, we have generated a strain of *P. denitrificans* Pd1222 in which subunit *ζ* has been deleted from the genome, and confirmed the absence of the *ζ* subunit from the mature ATP synthase complex. Surprisingly, we found that removing the *ζ* subunit does not substantially activate ATP hydrolysis. On this basis, we consider mechanisms of regulation and inhibition of ATP hydrolysis by ATP synthases in other species and discuss how the hydrolysis reaction may be blocked in the *P. denitrificans* enzyme even in the absence of the *ζ* subunit.

## 2. Results

### 2.1. Deletion of the *ζ* subunit from the *Paracoccus denitrificans* genome

The *ζ* gene was deleted by homologous recombination, using the same strategy as applied previously to delete the hydrogenase operon from *P. denitrificans* [31]. The protein is 104 residues long [29], and the *ζ* gene deletion (positions 29411 to 29725 on chromosome 2) removed all the coding 312 bp plus the stop codon from the DNA sequence. The strain with the *ζ* gene deleted is referred to hereon as the Δ*ζ* strain, for comparison with the strain containing the *ζ* gene referred to as the wild-type (WT) strain. Deletion of the *ζ* gene in the Δ*ζ* strain was confirmed as follows.

**Figure 2.**
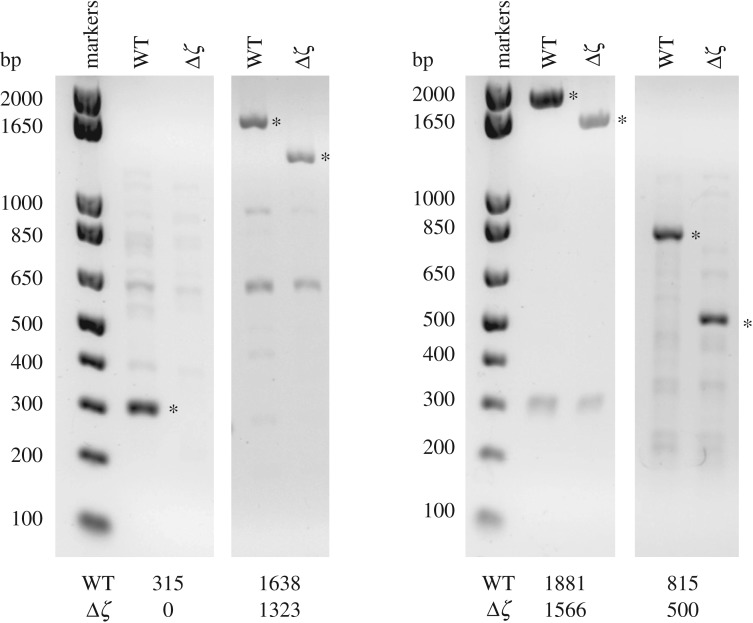
Genetic confirmation of the *ζ* knockout in *P. denitrificans*. PCR was used to amplify sequences of DNA that include the sequence for the *ζ* subunit (if present), and the products were analysed on 1% agarose gels (see electronic supplementary material, table S1 for the primers used). The expected lengths of the products from the WT and Δ*ζ* strains, which match the bands marked with asterisks, are shown at the bottom. In the leftmost reaction the primers bind to the coding sequence itself, so no product is observed from the Δ*ζ* strain; in the following three reactions the primers bind to the flanking regions, so the products from the Δ*ζ* strain are 315 bp shorter than from the WT.

First, PCR analyses were used to confirm the deletion genetically (figure 2). Colonies of the WT and Δ*ζ* strains were grown in LB media and analysed by PCR using four pairs of primers (see electronic supplementary material, table S1). The first pair of primer sequences are internal to the *ζ* gene so the WT colonies gave a product of length of 315 bp and the Δ*ζ* colonies gave no product. The other three pairs of primers bind to different sequences in the flanking regions on each side of the *ζ* gene so the WT strain gives products that are 315 bp longer than those from the Δ*ζ* strain. Finally, direct sequencing of the Δ*ζ* strain confirmed that the sequences on each side of the *ζ* deletion were joined correctly, with only the 315 bp from the *ζ* gene deleted.

**Figure 3.**
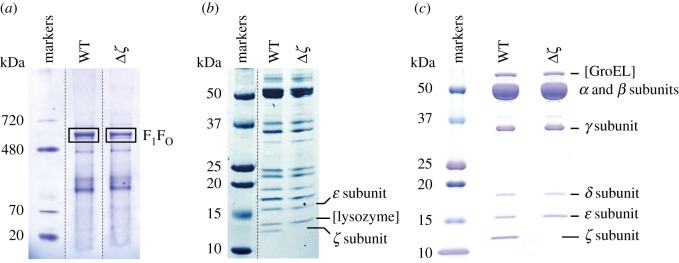
Protein confirmation of the *ζ* knockout in *P. denitrificans*. (*a*) BN-PAGE analyses of SBPs visualized using Coomassie R250. Orbitrap mass spectrometry analyses were performed on the F_1_F_O_ ATP synthase bands (shown by outline boxes) of both strains (see electronic supplementary material, table S2). (*b*) SDS-PAGE analyses of the ATP synthase bands excised from a BN-PAGE gel. (*c*) SDS-PAGE analyses of the F_1_-ATPase subcomplexes isolated from both strains. In (*b*) and (*c*) labelled bands were identified by MALDI mass spectrometry (see electronic supplementary material, tables S3 and S4).

Second, proteomic analyses were used to confirm the deletion on the protein level (figure 3). SBPs were prepared from both strains, then the proteins were solubilized using DDM detergent and analysed using Blue Native PAGE (figure 3*a*). The bands corresponding to the intact ATP synthase enzyme (expected molecular mass 546 kDa [13]) were excised and the presence of the ATP synthase subunits investigated by Orbitrap mass spectrometry. All the expected subunits were detected, except for the hydrophobic *c* subunit (which is typically difficult to detect). The *ζ* subunit was detected in the WT sample with a score considerably above the 95% confidence threshold, but not in the Δ*ζ* sample (see electronic supplementary material, table S2). Following a second Blue Native PAGE analysis, the bands were again excised from the gel, then analysed in a second dimension experiment by SDS-PAGE (figure 3*b*) to visualize the individual subunits. The banding patterns for the WT and Δ*ζ* samples match closely, except the protein with the smallest apparent mass of approximately 12 kDa, matching the expected 11 kDa mass of the *ζ* subunit [29], is absent from the Δ*ζ* sample. The three lowest molecular mass bands were excised from the gel and analysed by MALDI mass spectrometry, confirming the identity of the band absent from the Δ*ζ* sample as the *ζ* subunit (see electronic supplementary material, table S3). The F_1_-ATPase subcomplex was then isolated from both strains and analysed using SDS-PAGE (figure 3*c*). Again, the lowest molecular mass band, which is visually absent from the Δ*ζ* sample, was confirmed to contain the *ζ* subunit by MALDI mass spectrometry (see electronic supplementary material, table S4). Finally, the sequence of every remaining ATP synthase enzyme subunit in the Δ*ζ* strain was checked by direct sequencing of a set of PCR products and confirmed to be identical to in the WT strain. Therefore, there are no secondary mutations present in the variant strain that may affect catalysis and confound observations on the effects of deleting the *ζ* subunit.

In summary, a comprehensive set of analyses demonstrated that the *ζ* subunit of ATP synthase is not present in the Δ*ζ* strain.

### 2.2. Respiratory chain function in the WT and Δ*ζ* strains

Table 1 shows that the specific rates of NADH : O_2_ oxidoreduction by complexes I, III and IV (referred to as NADH oxidation), measured in the presence of the uncoupler gramicidin to dissipate the proton-motive force, are similar in the WT and Δ*ζ* strains. The rates observed here are higher than we reported previously for *P. denitrificans* SBPs [31] as a result of them being prepared by cell lysis in ultrapure water rather than in 10 mM Tris–SO_4_ (pH 7.4) buffer, and similar to the rate reported previously (1.8 µmol min^−1^ mg^−1^) by Zharova & Vinogradov [32]. Lysis in water gives a more highly inverted preparation and therefore fewer vesicles in which the complex I active site is occluded. Table 1 shows also that both the *P. denitrificans* and *E. coli* SBPs display considerably higher uncoupled rates of NADH oxidation than the SMPs prepared from bovine heart mitochondria. However, a substantial proportion of this difference arises simply from the lower molecular masses of the bacterial complexes (e.g. *P. denitrificans* complex I is around half the mass of the bovine enzyme). Therefore, the Δ*ζ* vesicles, like the WT vesicles, are fully competent for NADH oxidation.

**Table 1.** Specific activities for NADH oxidation in the vesicle systems studied. Measurements were carried out at 32°C in 10 mM Tris–SO_4_ (pH 7.4) and 250 mM sucrose, using 100 µM NADH (or 100 µM deaminoNADH for *E. coli*) with 8 µg ml^−1^ gramicidin used to dissipate Δp (Δp → 0) when required. The RCR value is the ratio of the rates in the presence and absence of gramicidin. DeaminoNADH precludes NADH oxidation by NDH2; background rates recorded in the presence of piericidin A (for NADH oxidation) were less than 5% of the measured rates and have been subtracted. See Material and methods for experimental details. The values are mean averages ± s.e.m. (*n* = 3).

	rate of reaction (µmol min^−1^ mg^−1^)		
species/strain	NADH oxidation	NADH oxidation (Δp → 0)	RCR for NADH oxidation
*Pd* wild-type	1.03 ± 0.04	2.15 ± 0.02	2.09 ± 0.09
*Pd* Δ*ζ* strain	1.23 ± 0.03	2.33 ± 0.03	1.90 ± 0.05
*B. taurus*	0.19 ± 0.01	0.72 ± 0.02	3.80 ± 0.06
*E. coli*	1.42 ± 0.03	1.67 ± 0.02	1.18 ± 0.03

### 2.3. ATP synthesis by SBPs from the WT and Δ*ζ* strains

**Figure 4.**
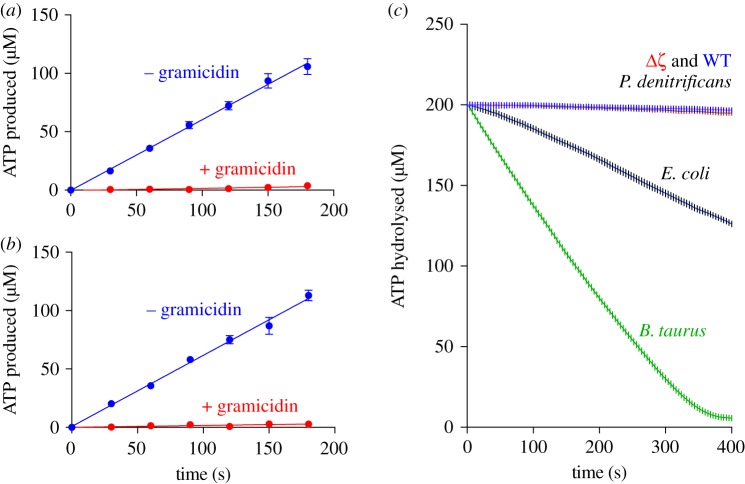
ATP synthesis driven by the NADH : O_2_ reaction in SBPs from the WT and Δ*ζ* strains and comparison of rates of ATP hydrolysis. (*a,b*) Examples of ATP synthesis data from SBPs from the WT (*a*) and Δ*ζ* (*b*) strains. Experimental data (blue) are compared with control data (red) recorded in the presence of 8 µg ml^−1^ gramicidin A to collapse Δp. Vesicles were supplied with 200 µM ADP and 2 mM Mg^2+^ and ATP synthesis was driven by using NADH oxidation to support Δp. (*c*) Examples of kinetic traces monitoring ATP hydrolysis by SBPs from the WT and Δ*ζ* strains of *P. denitrificans*, SBPs from *E. coli* and SMPs from *B. taurus*. ATP hydrolysis was conducted in 200 µM ATP and 2 mM Mg^2+^ and monitored using an ATP regenerating coupled assay system. See Material and methods for details.

To investigate whether ATP synthase in the Δ*ζ* strain is competent for ATP synthesis, NADH oxidation was used to create a proton-motive force to drive the ATP synthase to generate ATP. Figure 4 shows examples of data in which ATP synthesis was monitored over time by withdrawing aliquots from the reaction mixture and using the chemiluminescent luciferase assay to quantify the ATP concentrations. As described previously, ATP concentrations increase linearly throughout the experiment, and ATP production is fully sensitive to both addition of the uncoupler gramicidin (figure 4) and addition of the complex I inhibitor piericidin A [31]. Table 2 shows that the Δ*ζ* SBPs synthesize ATP at the same rate as the WT SBPs, so removing the *ζ* subunit has not affected ATP synthesis by the Δ*ζ* enzyme. Interestingly, SBPs from both strains of *P. denitrificans* produce ATP at much faster rates than SMPs prepared from bovine heart mitochondria and SBPs prepared from *E. coli*. Although exact comparisons are difficult because the relative amounts of the respiratory complexes may vary between the systems, the poor rate of ATP synthesis by the *E. coli* SBPs, despite their relatively high rates of NADH oxidation, can be attributed to them being poorly coupled, as reflected by their low RCR values (table 1). However, the same explanation does not apply to the bovine SMPs, indicating that caution should be used in correlating high respiratory control ratio (RCR) values with efficient coupling. In support of this observation, the *P. denitrificans* SBPs described by Zharova & Vinogradov [32] exhibited an RCR value of 6.4 for NADH oxidation and similar uncoupled rates to those reported here, but their rates of ATP synthesis were substantially less, only 0.38 µmol min^−1^ mg^−1^.

**Table 2.** Specific activities for ATP hydrolysis and ATP synthesis in the vesicle systems studied. Measurements were carried out at 32°C in 10 mM Tris–SO_4_ (pH 7.4) and 250 mM sucrose. ATP synthesis was conducted in 200 µM ADP and 2 mM Mg^2+^ and the inhibitor protein IF_1_ was present in assays on bovine SMPs. ATP hydrolysis vesicles was conducted in 200 µM ATP and 2 mM Mg^2+^. See Material and methods for further experimental details. The values reported are mean averages ± s.e.m. (*n* = 3).

species/strain	ATP hydrolysis (µmol min^−1^ mg^−1^)	ATP synthesis (µmol min^−1^ mg^−1^)	ratio of hydrolysis to synthesis
*Pd* wild-type	0.016 ± 0.002	1.39 ± 0.11	0.015 ± 0.002
*Pd* Δ*ζ* strain	0.026 ± 0.002	1.40 ± 0.05	0.014 ± 0.001
*B. taurus*	1.24 ± 0.01	0.33 ± 0.01	3.8 ± 0.1
*E. coli*	0.38 ± 0.01	0.19 ± 0.03	2.0 ± 0.3

### 2.4. ATP hydrolysis by SBPs from the WT and Δ*ζ* strains

Table 2 shows that SBPs from the Δ*ζ* strain hydrolyse ATP faster than SBPs from the WT strain, but that the increase in rate is only moderate (1.6-fold). Both rates observed are consistent with that reported previously by Zharova & Vinogradov [32], and extremely low in comparison with those of bovine SMPs. When the rates are reported relative to the rates of ATP synthesis, the difference between the systems is even more striking. Therefore, removing the *ζ* subunit from the *P. denitrificans* ATP synthase has not substantially activated it to catalyse ATP hydrolysis.

It has been reported that the detergent lauryldimethylamine oxide (LDAO) [30] and the oxyanion sulfite (SO_3_^2−^) [33] increase the rate of hydrolysis by *P. denitrificans* ATP synthase. The data in table 3 confirm that both LDAO and sulfite increase the rates of hydrolysis substantially. However, the rates of both the WT and the Δ*ζ* strains increase similarly, leaving the ratio between them essentially unaffected. We were unable to activate ATP hydrolysis by catalysing synthesis beforehand, as has been reported previously [32]. Table 3 reports two sets of hydrolysis data for the *P. denitrificans* SBPs, recorded with different concentrations of ATP and Mg^2+^. Our initial data were recorded using 200 µM ATP and 2 mM Mg^2+^, our standard condition transferred from earlier SMP studies [34,35], whereas others have reported a requirement for much higher Mg-ATP concentrations based on the relatively high *K*_M_ value of 280 µM they observed [33]. Here, we found that the *K*_M_ value for ATP is strongly dependent on the concentration of Mg^2+^. We measured values of 84 ± 8 µM for the WT strain and 63 ± 7 µM for the Δ*ζ* strain in 5 mM Mg^2+^, so that the second set of values we report, recorded using 2.5 mM ATP and 2.5 mM Mg^2+^, are only moderately higher than the first, and they display the same characteristics. Therefore, the similar increases observed for both the WT and Δ*ζ* strains suggests that LDAO and sulfite activate hydrolysis by a mechanism that is independent of the presence or absence of the *ζ* subunit.

**Table 3.** The activation of ATP hydrolysis by SBPs from the wild-type and Δ*ζ* strains of *P. denitrificans* using LDAO and/or sulfite. Measurements were carried out at 32°C in 10 mM Tris–SO_4_ (pH 7.4) and 250 mM sucrose, using 200 µM ATP and 2 mM Mg^2+^ for the standard condition and 2.5 mM ATP : Mg for the high ATP condition; 0.4% LDAO and/or 10 mM sulfite were added as indicated. See Material and methods for further experimental details. The values reported are mean averages ± s.e.m. (*n* = 3).

	rate of ATP hydrolysis (µmol min^−1^ mg^−1^)		
condition	wild-type	Δ*ζ*	ratio
no addition	0.016 ± 0.002	0.026 ± 0.002	1.6 ± 0.2
LDAO	0.192 ± 0.002	0.373 ± 0.008	1.9 ± 0.0
sulfite	0.097 ± 0.005	0.168 ± 0.003	1.7 ± 0.0
LDAO + sulfite	0.276 ± 0.005	0.374 ± 0.002	1.4 ± 0.0
no addition, high ATP	0.021 ± 0.002	0.020 ± 0.001	1.0 ± 0.1
LDAO, high ATP	0.588 ± 0.018	1.113 ± 0.180	1.9 ± 0.3
sulfite, high ATP	0.168 ± 0.005	0.254 ± 0.013	1.5 ± 0.1
LDAO + sulfite, high ATP	0.301 ± 0.005	0.345 ± 0.011	1.1 ± 0.0

### 2.5. Rates of ATP hydrolysis by the F_1_-ATPase subcomplex from the WT and Δ*ζ* strains

The results described above are in stark contrast to the data measured by Zarco-Zavala and co-workers [30], who observed that the ATPase activity of *Pd*-F_1_-ATPase (in the presence of 60 mM sodium sulfite) increased substantially upon removal of the endogenous *ɛ* and *ζ* subunits by immunoaffinity chromatography. To investigate whether the different behaviour could arise from a difference between the intact ATP synthase present in our SBPs and the F_1_-ATPase investigated by Zarco-Zavala and co-workers [30], we measured ATP hydrolysis by the F_1_-ATPases prepared from both the WT and Δ*ζ* strains. Table 4 shows that the small difference between the WT and the Δ*ζ* strains observed in SBPs is maintained in the F_1_-ATPase, and that the stimulation of the rate observed with LDAO and sulfite is again independent of the presence of the *ζ* subunit. Therefore, the different behaviour observed does not arise from differences between the intact enzyme and the F_1_ subcomplex.

**Table 4.** The activation of ATP hydrolysis by the purified F_1_-ATPase subcomplex from the wild-type and Δ*ζ* strains of *P. denitrificans* using LDAO and/or sulfite. Measurements were carried out at 32°C in 10 mM Tris–SO_4_ (pH 7.4) and 250 mM sucrose, using 200 µM ATP, in the presence of 0.4% LDAO and/or 10 mM sulfite as indicated. See Material and methods for further experimental details. The values reported are mean averages ± s.e.m. (*n* = 3).

	rate of ATP hydrolysis (µmol min^−1^ mg^−1^)		
condition	wild-type	Δ*ζ*	ratio
no addition	0.024 ± 0.004	0.047 ± 0.007	2.0 ± 0.4
LDAO	1.61 ± 0.03	4.25 ± 0.04	2.6 ± 0.1
sulfite	2.52 ± 0.06	3.92 ± 0.05	1.6 ± 0.0
LDAO + sulfite	3.81 ± 0.05	5.12 ± 0.06	1.3 ± 0.0

## 3. Discussion

The biochemical work of García-Trejo and co-workers [29,30,36], together with the structure of *P. denitrificans* ATP synthase that revealed the *ζ* subunit bound in a manner analogous to the eukaryotic inhibitor protein IF_1_ [13], indicated that the *ζ* subunit is responsible for preventing ATP hydrolysis and enforcing unidirectional catalysis in the *P. denitrificans* enzyme. However, deleting the *ζ* subunit caused only very moderate increases in ATP hydrolysis (less than twofold), and the rates remain very low in comparison with the rates observed from the mammalian and *E. coli* enzymes (table 2). Therefore, we conclude that removal of the *ζ* subunit is not sufficient to activate ATP hydrolysis in *P. denitrificans* ATP synthase.

Although the *ζ* subunit in *P. denitrificans* adopts a similar binding mode to the IF_1_ inhibitor proteins of eukaryotic ATPases, the *ζ* subunit is generally considered to be a permanently bound subunit, whereas IF_1_ is released from the eukaryotic enzyme when it rotates in the synthesis direction [9]. This raises the intriguing question of how the *P. denitrificans* enzyme synthesizes with the *ζ* subunit present. The *ζ* subunit is 104 residues long, whereas only residues 1–32 (which form the inhibitory helix) and residues 82–103 (which form helix 4) have been resolved structurally in the ATP synthase complex [13]. The structure of the *ζ* subunit in solution showed that, while residues 1–18 were unstructured, residues 19–103 formed a four-helix bundle [37] and in the structure of *P. denitrificans* ATP synthase, helix 4 was observed to interact with one of the α-subunits in F_1_. It is possible that the *N*-terminal helix of the *ζ* subunit is ejected from its inhibitory site in F_1_ during synthesis, but remains bound to the complex through interactions of the four-helix bundle, to snap back into place during hydrolysis, in the same mode of action as exhibited by the eukaryotic inhibitor proteins.

Our conclusion that removing the *ζ* subunit is not sufficient to activate ATP hydrolysis in *P. denitrificans* ATP synthase appears to contrast with the results of García-Trejo and co-workers. However, we deleted only the *ζ* subunit, whereas the chromatography procedure employed by García-Trejo and co-workers, which provided a much greater activation of the hydrolysis rate, removed both the *ɛ* and *ζ* subunits [29]. Thus, the difference may be due to the additional removal of the *ɛ* subunit. Importantly, deleting the *ζ* subunit had no observable effect on the stability of the *P. denitrificans* ATP synthase, on its ability to synthesize ATP nor on the growth of *P. denitrificans* cells (see electronic supplementary material, figure S1). García-Trejo and co-workers observed the effects of removing both the *ɛ* and *ζ* subunits in the F_1_ domain, not in the intact enzyme, in which more subtle approaches would be required in order to retain the essential structural and functional roles of the *ɛ* subunit in connecting the central stalk to the membrane *c*-ring motor [1].

It is possible that, in the absence of the *ζ* subunit, the C-terminus of the *P. denitrificans ɛ* subunit adopts an altered, inhibitory conformation, like that observed in the *E. coli* enzyme [14,21], and blocks hydrolysis in its place. In the structure of *P. denitrificans* ATPase the C-terminal domain (65 residues) of the *ɛ* subunit, which is predicted to be predominantly helical, was not resolved [13] and sequence comparisons reveal only limited homology between the *ɛ* subunit C terminii in *P. denitrificans*, *B. taurus*, *E. coli* or *C. thermarum*. However, key residues that coordinate an ATP molecule that is bound to the *ɛ* subunit in *E. coli* and *C. thermarum* and linked to regulation of the *E. coli* enzyme [14,17,19] are absent from *P. denitrificans*, and it is important to note that the *ζ*-free *P. denitrificans* enzyme catalyses ATP hydrolysis much more slowly than the ɛ-regulated *E. coli* enzyme, matching much more closely the characteristics of the unidirectional *C. thermarum* enzyme for which structural data has suggested hydrolysis is not blocked by the *ɛ* subunit [15]. Establishing whether the C-terminus of the *ɛ* subunit does play a role in blocking hydrolysis in the *ζ*-free *P. denitrificans* enzyme will require further genetic and structural work. Alternatively, hydrolysis by the *P. denitrificans* enzyme may be prevented by formation of a stable ADP-bound state [25–27], from which the enzyme cannot escape in the hydrolysis direction. This mechanism has been proposed for the *C. thermarum* enzyme [15] and also for the WT *P. denitrificans* enzyme by Zharova & Vinogradov [38], and it is supported by activation of the *P. denitrificans* enzyme by LDAO, which has been suggested to occur by relieving ADP inhibition [39].

Finally, it is interesting to consider the role of ratchet inhibitory mechanisms for ATP hydrolysis in the evolution of ATP synthases. The most efficient energy-conserving catalysts are thermodynamically reversible: they switch immediately from one direction of catalysis to the other across the equilibrium position, and catalysis in either direction is substantial, with only a small displacement from the equilibrium position [40]. Enzymes with ratchet *in vivo* mechanisms (from yeast, chloroplasts and *E. coli*) have been shown to catalyse reversibly and efficiently *in vitro* [41,42]. By contrast, *P. denitrificans* ATP synthase (in the presence of the *ζ* subunit) does not catalyse reversibly [28]. It is easy to see why evolutionary drivers may have acted to increase the efficiency of ATP synthases, perhaps by decreasing activation barriers for ADP release. However, if the uncontrolled ‘reverse’ hydrolysis reaction is deleterious the most efficient ATP synthase is not necessarily the most biologically effective. Ratchet mechanisms may provide a method to navigate the evolution of efficient ATP synthase enzymes by selectively inhibiting hydrolysis and protecting against the side effects of increased efficiency.

## 4. Material and methods

### 4.1. Generation of the Δ*ζ* strain of *Paracoccus denitrificans*

The Δ*ζ* strain was created in the Δhydrogenases strain of *P. denitrificans* Pd1222 described previously [31] that is referred to here as the WT strain. The same strategy as used previously [31] was used to create an unmarked deletion of the *ζ* gene (Pden_2862) by homologous recombination. A deletion cassette, containing two sequences homologous to regions on each side of the *ζ* gene followed by the kanamycin resistance gene (*kan^R^*) was assembled by Gibson assembly and placed into the EcoR1 site of the *lacZ*-containing pRVS1 plasmid. The plasmid was transformed into the MFD*pir* strain of *E. coli* (to avoid mobilizing *E. coli* genes [31,43]) and conjugated into the WT strain of *P. denitrificans*. The resulting cells were plated onto kanamycin (100 µg ml^−1^) to identify colonies that had undergone the first recombination event [31,44]. Then, positive colonies were plated onto X-gal (200 µg ml^−1^) and white colonies, which have also undergone the second recombination event, were selected. The absence of *kan^R^* and the plasmid, as well as of the *ζ* gene (nt 29411–29725 of chromosome 2), was confirmed by sequencing and sensitivity to kanamycin.

### 4.2. Preparation of inverted membrane vesicles

*Paracoccus denitrificans* sub-bacterial particles (SBPs) were prepared as described previously [31] except that cell lysis was carried out in ultrapure water instead of in 10 mM Tris–SO_4_ buffer. Briefly, cells were grown aerobically at 30°C and 225 rpm and harvested at mid-exponential phase by centrifugation. The following steps were performed at 4°C. The cell pellets were resuspended in 10 mM Tris–SO_4_ (pH 7.4) and 150 mM NaCl, recentrifuged, and then resuspended in 10 mM Tris–SO_4_ (pH 7.4) and 500 mM sucrose to an OD_600_ of approximately 7.5. Hen egg-white lysozyme (Sigma, 0.25 mg ml^−1^) was added, the suspension was incubated for 60 min, then the digested cells were collected by centrifugation. The pellet was resuspended in ultrapure water to initiate cell lysis, then 5 mM MgSO_4_ and a few flakes of bovine pancreatic DNase (Sigma) were added, and the lysate centrifuged twice to remove debris. Finally, the supernatant was centrifuged to pellet the SBPs, the sample resuspended to approximately 10 mg ml^−1^ in 5 mM Tris–SO_4_ (pH 7.4) and 250 mM sucrose, and frozen at −80°C until required. Submitochondrial particles (SMPs) from *B. taurus* heart mitochondria were prepared as described previously [34]. *E. coli* SBPs were prepared from the *E. coli* BL21 (DE3) strain (New England Biolabs Inc.) as described previously [31].

### 4.3. Purification of F_1_-ATPase from *Paracoccus denitrificans*

The *P. denitrificans* F_1_-ATPase subcomplex was prepared at room temperature using a method based on that described by Morales and co-workers [45]. Five milliliters of chloroform (pre-equilibrated against 1 M Tris–HCl, pH 7.4) were added to a 10 ml suspension of approximately 10 mg ml^−1^ SBPs in 5 mM Tris–SO_4_ (pH 7.4) and 250 mM sucrose. The two phases were mixed vigorously for 20 s, then separated by centrifugation (5000*g*, 5 min). The upper aqueous phase was removed and centrifuged (16 000*g*, 60 min) to remove insoluble debris. A stream of N_2_ was used to remove the chloroform then the sample was applied to a 1 ml HiTrap-Q HP column (GE Healthcare Life Sciences) pre-equilibrated in buffer containing 50 mM Tris–HCl (pH 7.4), 10% (v/v) glycerol, 0.5 mM ATP, 2 mM MgCl_2_ and the Roche cOmplete, EDTA-free protease-inhibitor cocktail. The column was washed with 5 ml of buffer, then proteins were eluted with a 15 ml linear gradient from 0 mM to 200 mM NaCl. The F_1_-ATPase eluted at 80–115 mM NaCl. Fractions were analysed by SDS-PAGE, pooled and concentrated, then applied to a Superdex 200 gel filtration column (GE Healthcare Life Sciences) pre-equilibrated in the same buffer. The F_1_-ATPase subcomplex eluted in the second major peak. Fractions were pooled, concentrated to approximately 1 mg ml^−1^, and frozen at –80°C until required.

### 4.4. Kinetic activity assays

NADH and deaminoNADH oxidation and ATP hydrolysis were measured at 32°C, in 20 mM Tris–SO_4_ (pH 7.45) and 250 mM sucrose, using a Molecular Devices SpectraMax Plus 96-well microplate reader. Oxidation of 100 µM NADH or deaminoNADH were measured directly at 340–380 nm (*ɛ* = 4.81 mM^−1^ cm^−1^). Hydrolysis of ATP (typically 200 µM) was measured using a coupled assay system to detect the production of ADP [34,46], with 2.5 µM piericidin A to prevent complex I oxidizing the NADH required by the coupled assay system. For *E. coli* SBPs, all ATP hydrolysis measurements were performed using deaminoNADH (which was confirmed to react equivalently to NADH in the coupled assay system) to also preclude reoxidation of NADH by alternative NADH : quinone oxidoreductases. The reactions were monitored spectroscopically via the absorbance of NADH, and 8 µg ml^−1^ gramicidin was used to dissipate Δp when required.

ATP synthesis was measured in buffer containing 20 mM Tris–SO_4_ (pH 7.45), 250 mM sucrose, 200 µM ADP, 10 mM KPO_4_, 2 mM MgSO_4_, 40 µM diadenosine pentaphosphate (AP5A, to inhibit adenylate kinase activity), 0.9 µM of the bovine ATP synthase inhibitor protein IF_1_ (for experiments with SMPs, a truncated (amino acids 1–60) hexahistidine-tagged form prepared as described previously [8]) and typically 30 µg ml^−1^ of membrane vesicles. The reaction was initiated by addition of 200 µM NADH, and NADH oxidation followed spectrophotometrically to confirm its rate as constant and within the expected range. ATP production was monitored by withdrawing and quenching 10 µl aliquots of reaction mixture, starting immediately and then at intervals throughout the experiment. Each 10 µl aliquot was added immediately to 40 µl of 4% trifluoroacetic acid, then 20 s later 950 µl of neutralizing buffer (1 M Tris–SO_4_, pH 8.1) were added. ATP concentrations in the quenched aliquots were determined using the Roche ATP Bioluminescence Assay Kit CLS II in a Berthold Autolumat tube luminometer, by comparison with known standards.

### 4.5. Electrophoresis

Agarose (BioGene Ltd) gels were prepared in 100 mM Tris (pH 8), 100 mM boric acid and 2 mM EDTA (TBE buffer) containing 100 ng ml^−1^ of UltraPure ethidium bromide (ThermoFisher Scientific) and typically 1.0% (w/v) agarose. Samples were loaded in DNA gel loading dye (Invitrogen), alongside the 1 Kb plus DNA ladder (Invitrogen), run at 100 V for 1 h and visualized using ethidium bromide fluorescence on a ChemiDoc MP System (Bio-Rad).

SDS-PAGE analyses were performed using either Novex WedgeWell 10–20% tris–glycine gels or Novex 10–20% tris–glycine gels. Proteins were reduced with 100 µM DTT then approximately 10 µg of protein loaded per well, alongside the Precision Plus Protein Kaleidoscope prestained protein standards (Bio-Rad), and visualized using Coomassie R250. Blue native polyacrylamide gel electrophoresis (BN-PAGE) was performed using NativePAGE Novex 3–12% Bis-Tris gels (Invitrogen). Vesicles were solubilized using a 2 : 1 DDM : protein ratio, and approximately 8 µg samples loaded in each well alongside the NativeMark Protein Standard (Invitrogen). Gels were run as described previously [47] and visualized using Coomassie R250.

### 4.6. Proteomic analyses

For mass spectrometry analyses, bands were excised from SDS-PAGE or BN-PAGE gels, and digested with trypsin as described previously [47]. The tryptic digests were analysed by either matrix-assisted laser-desorption ionization (MALDI), using an Applied Biosystems/MDS SCIEX model 4800 Plus MALDI–TOF-TOF spectrometer, or separated by LC-MS and analysed using an Orbitrap Q-Exactive mass spectrometer, as described previously [47]. Spectra were assigned to peptide sequences and proteins identified using the Mascot 2.4 application (Matrix Science Ltd) [48] to search the National Centre for Biotechnology Information, non-redundant-protein database (NCBInr, v. 11 June 2012). Peptide precursor mass tolerances of 5 ppm and 70 ppm, and fragment mass tolerances of 0.01 and 0.8 Da were allowed for Orbitrap and MALDI analyses, respectively, allowing for one missed cleavage, plus methionine oxidation and cysteine propionamide formation as variable modifications.

## Supplementary Material

Supplementary Tables 1 to 4 and Figure 1

## Data accessibility

All relevant data are included in the manuscript and the electronic supplementary material.
