# Supplementary material for: Deleting the IF1-like ζ subunit from Paracoccus denitrificans ATP synthase is not sufficient to activate ATP hydrolysis
Source: Open Biol. 2018 Jan 24;8(1):170206. doi: 10.1098/rsob.170206 (PMC5795051; doi:10.1098/rsob.170206)
Supplement: Supplementary Tables 1 to 4 and Figure 1 [file rsob170206supp1.pdf]

Supplementary Data

Supplementary Table 1

Primer	Sequence	Product length (bp)	
		WT	Δζ
a1 (sense)	ATGACCACATTTGACGACCG	315	0
a2 (antisense)	TCAGATCTCGCTGATGATCT		
b1 (sense)	ATGAACATGGAGAGCTTGTC	1638	1323
b2 (antisense)	GATGCGAAACGCCCCGGATT		
c1 (sense)	AAGGTGACAATCTTTATGCG	1881	1566
c2 (antisense)	GACCAGCGCCAGAAGCTTTT		
d1 (sense)	CGGTGGCGTCATCCTTGAAA	815	500
d2 (antisense)	GACGGCATCTTCATCGGCGC		

The sequences of the primers used to confirm deletion of the ζ gene and the predicted lengths of the PCR products.

Supplementary Table 2

ATP synthase subunit	WT			Δζ		
	Score / 44	Coverage	Peptides	Score / 45	Coverage	Peptides
α	6242	71	39	4606	70	37
β	6972	85	32	6361	85	31
γ	2411	77	24	1608	77	24
δ	842	66	13	735	66	13
ε	424	51	7	379	51	7
b	343	48	13	373	45	10
b’	1514	31	7	1099	30	6
a	179	14	3	110	10	2
c	n. d.	n. d.	n. d.	n. d.	n. d.	n. d.
ζ	193	55	5	n. d.	n. d.	n. d.

Summary of the Orbitrap analyses of bands excised from the BN-PAGE gel shown in Figure 3A. Mascot scores for each identification are presented, in comparison to the $p < 0.05$ Ions score cut-off for each experiment. Coverage is the percentage of the protein sequence covered by detected peptides. Peptides are the number of unique peptides detected.

Supplementary Table 3

Protein	WT			Δζ		
	Score	Coverage	Peptides	Score	Coverage	Peptides
ε subunit	336 /55	50	5	322 /54	50	5
lysozyme	n. d.	n. d.	n. d.	101 /56	21	2
ζ subunit	362 /54	42	4	n. d.	n. d.	n. d.

Summary of the MALDI analyses of low molecular weight bands excised from the SDS-PAGE gel shown in Figure 3B. Mascot scores for each identification are presented, in comparison to the $p < 0.05$ Ions score cut-off for each experiment. Coverage is the percentage of the protein sequence covered by detected peptides. Peptides are the number of unique peptides detected.

Supplementary Table 4

	WT			Δζ		
Protein	Score	Coverage	Peptides	Score	Coverage	Peptides
GroEL	460 /55	20	6	502 /55	24	7
α	475 /56	23	9	720 /56	21	8
β	411 /55	21	6	496 /56	24	7
γ	723 /55	50	9	683 /55	38	8
δ	92 /54	9	2	202 /55	27	4
ε	114 /54	35	3	204 /53	40	4
ζ	597 /55	61	7	n. d.	n. d.	n. d.

Summary of MALDI analyses of bands excised from the SDS-PAGE gel shown in Figure 3C. Mascot scores for each identification are presented, in comparison to the $p < 0.05$ Ions score cut-off for each experiment. Coverage is the percentage of the protein sequence covered by detected peptides. Peptides are the number of unique peptides detected (n. d., not detected).

Supplementary Figure 1

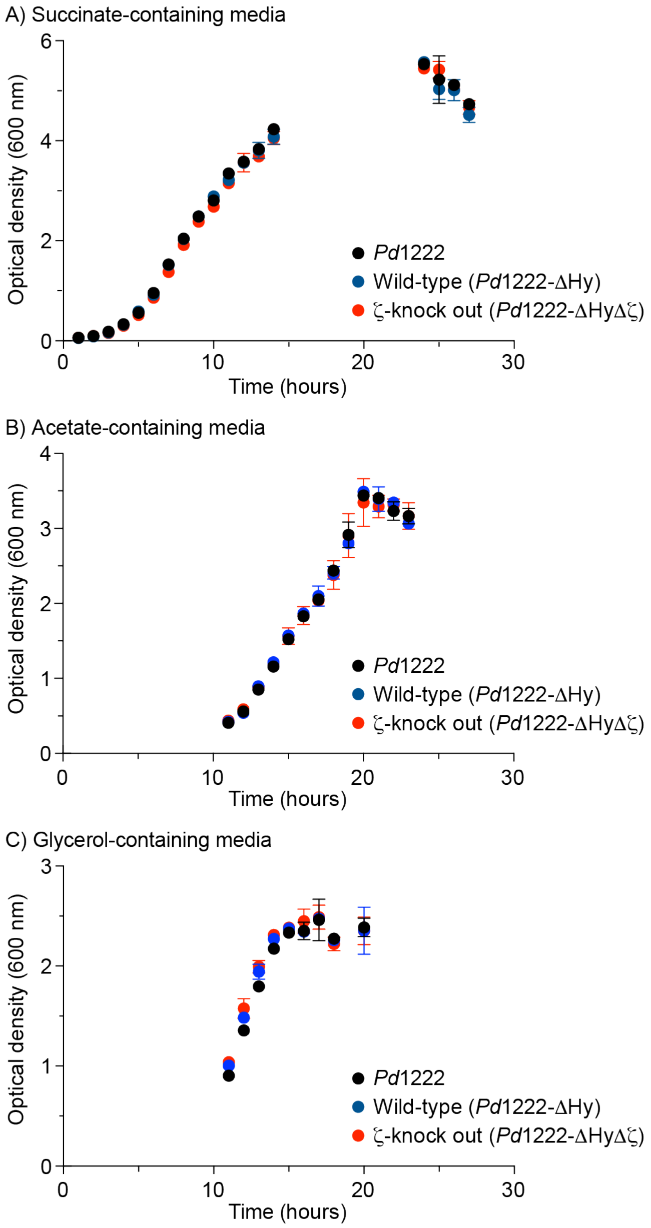

Comparison of growth curves for *P. denitrificans* cells from the parent *Pd*1222 strain, the Δhydrogenases strain referred to here as the wild-type, and the ζ-knock out strain. Cells were grown in well-aerated minimal media (pH 7.2) containing either 50 mM succinate, acetate or glycerol at 30 ˚C. Experiments were initiated using a freshly-grown stationary-phase starter culture added to a starting optical density of 0.05 and carried out in triplicate. The minimal media contained 9.35 mM NH_4Cl, 2 mM $MgSO_4$, 0.07 mM $CaCl_2$, 0.29 mM KH_2PO_4, 0.69 mM K_2HPO_4, 25.2 mM Na-HEPES, 19.6 μM Na_2-EDTA, 9 μM $FeSO_4$, 0.1 μM $MnCl_2$, 0.8 μM $CuCl_2$, 1 μM Na_2MoO_4 and 2.5 μM $ZnCl_2$.
